# DNA damage and expression of DNA methylation modulators in urine-derived cells of patients with hypertension and diabetes

**DOI:** 10.1038/s41598-020-60420-9

**Published:** 2020-02-25

**Authors:** Akihito Hishikawa, Kaori Hayashi, Norifumi Yoshimoto, Ran Nakamichi, Koichiro Homma, Hiroshi Itoh

**Affiliations:** 1 Division of Nephrology, Endocrinology and Metabolism, Department of Internal Medicine, Keio University School of Medicine, Tokyo, Japan; 2 Department of Emergency Medicine, School of Medicine, Keio University, Tokyo, Japan

**Keywords:** Biomarkers, Nephrology

## Abstract

Diabetes and hypertension have become the primary causes of chronic kidney disease worldwide. However, there are no established markers for early diagnosis or predicting renal prognosis. Here, we investigated the expression profiles of DNA repair and DNA methylation factors in human urine-derived cells as a possible diagnostic or renal prognosis-predicting marker. A total of 75 subjects, aged 63.3 ± 1.9 years old, were included in this study. DNA and RNA were extracted from 50 mL of urine samples. We evaluated DNA double-strand breaks (DSBs) by the quantitative long distance-PCR method and performed real-time RT-PCR analysis to analyze the expression of renal cell-specific markers, DNA DSB repair factor KAT5, DNA methyltransferases DNMTs, and demethylation enzymes TETs. In patients with hypertension and diabetes, DNA DSBs of the nephrin gene increased with decreased urine KAT5/nephrin expression, consistent with our previous study (Cell Rep 2019). In patients with hypertension, DNA DSBs of the AQP1 gene were increased with elevated urine DNMTs/AQP1 and TETs/AQP1 expression. Moreover, urine DNMTs/AQP1 expression was significantly correlated with the annual eGFR decline rate after adjustment for age, baseline eGFR, the presence of diabetes and the amount of albuminuria, suggesting a possible role as a renal prognosis predictor.

## Introduction

The prevalence of chronic kidney disease (CKD) has increased globally and become recognized as a worldwide health problem^1^. Specifically, the number of CKD caused by diabetes or hypertension is constantly growing along with rapid aging of the world population.

The DNA damage repair system is indispensable for maintaining genome integrity, and accumulation of DNA damage is linked to aging and age-related diseases. Recently, we have reported a possible association of KAT5 (lysine acetyltransferase 5, Tip60)-mediated DNA damage repair and DNA methylation changes in kidney glomerular podocytes^2^. KAT5, a repair factor of DNA double-strand breaks (DSBs), is crucial for podocyte maintenance, and deletion of podocyte KAT5 caused an increase in DNA DSBs together with increased DNA methylation and expression of DNA methyltransferase 1 (DNMT1) and DNMT3B. Reduced KAT5 expression was observed in the glomeruli of diabetic nephropathy in mouse models and humans. However, the association of KAT5 expression with DNA DSB levels and kidney disease progression has not been clarified.

A kidney biopsy is an invasive procedure that is not often performed in patients with an early stage of hypertension or diabetes. In addition, many histological findings of hypertensive nephrosclerosis and diabetic kidney disease are common and coexist; therefore, it is hard to determine which pathophysiology plays a dominant role in patients with both hypertension and diabetes. Urine examinations are noninvasive procedures, and because they identify changes in gene expression in urine-derived cells^3–6^, they may be particularly useful for the noninvasive assessment of kidney condition and prediction of renal outcomes in early hypertension and diabetes.

There are several methods to detect DNA DSBs. The most commonly used DNA DSB marker is phosphorylated histone H2AX (γH2AX), which plays an important role in the recruitment of DNA repair factors to damaged sites^7^. In the present study, we performed the quantitative long-distance PCR method using DNA samples of urine-derived cells to estimate kidney DNA DSBs because PCR analysis can be performed with small amounts of DNA. The quantitative long-distance PCR method for detecting DNA damage is based on the assumption that DNA with fewer damage lesions will amplify to a greater extent than more damaged DNA if equal amounts of DNA from different samples are amplified under identical conditions^8,9^.

The aim of this study was to evaluate kidney DNA damage and repair in patients with hypertension and/or diabetes, investigating the amount of DNA DSBs by the quantitative long-PCR analysis and gene expression of a DNA repair factor, KAT5, and related DNA methylation modulators in human urine-derived cells.

## Results

### Participant characteristics

Individuals who visited the outpatient Department of Nephrology at the Keio University Hospital from May 1, 2018 to June 30, 2019, were enrolled. A total of 75 participants (47 males, 28 females) aged 63.3 ± 1.9 years old were eligible for this study. Table 1 shows the general characteristics of the subjects. In subjects with hypertension, age and systolic BP were significantly higher.

**Table 1:** Participant characteristics.

	Total	Group A HT(−) DM(−)	Group B HT(+) DM(−)	Group C HT(+) DM(+)	p value A vs B	p value B vs C	p value A vs C
N (%)	75 (100)	19 (25)	41 (55)	15 (20)			
Age (y)	63.3 ± 1.9	50.7 ± 3.4	66.2 ± 2.3	71.3 ± 3.9	**0.0004**	0.2969	**0.0002**
Sex (male) (%)	47 (63)	10 (53)	27 (66)	10 (67)	0.3271	0.9546	0.4090
BMI (kg/m^2^)	23.1 ± 0.4	21.8 ± 1.0	23.1 ± 0.5	24.5 ± 1.0	0.2456	0.1834	**0.0445**
Systolic BP (mmHg)	135.3 ± 2.3	111.6 ± 4.1	138.7 ± 2.3	146.4 ± 3.8	**<0.0001**	0.0860	**<0.0001**
Diastolic BP (mmHg)	78.4 ± 1.7	69.1 ± 3.9	80.5 ± 2.2	80.7 ± 3.6	**0.0124**	0.9630	**0.0316**
eGFR (ml/min/1.73m^2^)	65.3 ± 2.5	81.7 ± 4.6	59.6 ± 3.0	61.9 ± 4.9	**0.0001**	0.6862	**0.0044**
Urinary protein (g/g Cr)	0.2780.13	0.031 ± 0.26	0.43 ± 0.18	0.17 ± 0.30	0.2145	0.4475	0.7357
Albumin-to-creatinine ratio (ACR) (mg/g Cr)	156.3 ± 73.7	9.0 ± 146.4	251.2 ± 99.7	83.7 ± 164.8	0.1758	0.3871	0.7360
Glucose (mg/dl)	110.7 ± 4.9	87.4 ± 10.3	106.9 ± 5.9	140.9 ± 9.6	0.1041	**0.0036**	**0.0003**
HbA1c (%)	5.7 ± 0.1	5.4 ± 0.1	5.6 ± 0.1	6.6 ± 0.1	**0.0412**	**<0.0001**	**<0.0001**
Dislipidemia (%)	39 (52)	2 (11)	25 (61)	12 (80)	**0.0003**	0.1830	**<0.0001**
Hyperuricemia (%)	28 (37)	0 (0)	20 (49)	8 (53)	**0.0002**	0.7628	**0.0003**

Group A: patients without hypertension and diabetes, Group B: patients with hypertension, Group C: patients with hypertension and diabetes. HT, hypertension; DM, diabetes mellitus; BMI, body mass index; BP, blood pressure; eGFR, estimated glomerular filtration rate; HbA1c, hemoglobin A1c.

### Detection percent of gene expression of cell markers and epigenetic modifiers

We obtained urine pellet RNA and performed real-time RT-PCR analysis as described in the **Methods** section. The detection percent of all the cell markers and the epigenetic modifiers analyzed in this study is shown in Supplementary Table 1.

### DNA double-strand breaks (DSBs) of the nephrin gene and urine KAT5/nephrin expression

**Figure 1:**
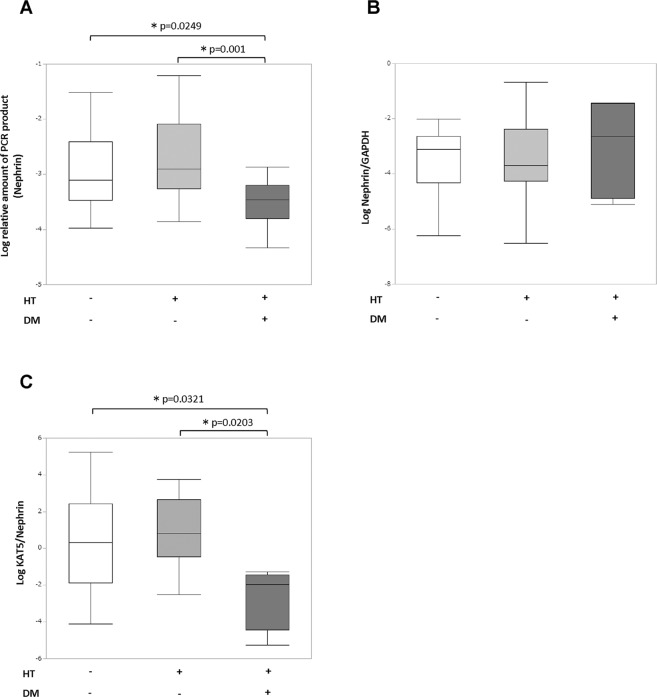
DNA double-strand breaks (DSBs) of the nephrin gene and urine KAT5/nephrin expression. (**A**) Detectable increase in DNA DSB sites in the nephrin gene by the real-time RT-PCR method in patients with diabetes and hypertension compared with controls or patients with hypertension alone. (**B**) Real-time RT-PCR analysis of nephrin expression. (**C**) Real-time RT-PCR analysis of urine KAT5/nephrin expression. DM, diabetes mellitus; HT, hypertension.

DNA DSBs of each cell marker gene were evaluated by the quantitative long-distance PCR method as described in the **Methods** section. In patients with diabetes and hypertention, the long-PCR product of the nephrin gene that is expressed specifically in podocyte was decreased compared with controls or patients with hypertension alone, which showed promoted DNA DSBs of the nephrin gene in podocytes (Fig. 1A). The expression of nephrin gene itself did not differ between each group (Fig. 1B). We did not isolate podocytes from the urine-derived cells; instead we evaluated KAT5 expression of podocytes by correcting the KAT5 expression with nephrin expression which is a podocyte specific cell marker. Urine KAT5/nephrin was decreased in patients with both hypertension and diabetes compared with controls or patients with hypertension alone (Fig. 1C). These results are compatible with our previous research that showed that DNA DSBs are promoted with decreased KAT5 expression in diabetic nephropathy podocytes.

### DNA double-strand breaks of the AQP1 gene and expression of DNA methylation modifiers correction for AQP1

**Figure 2:**
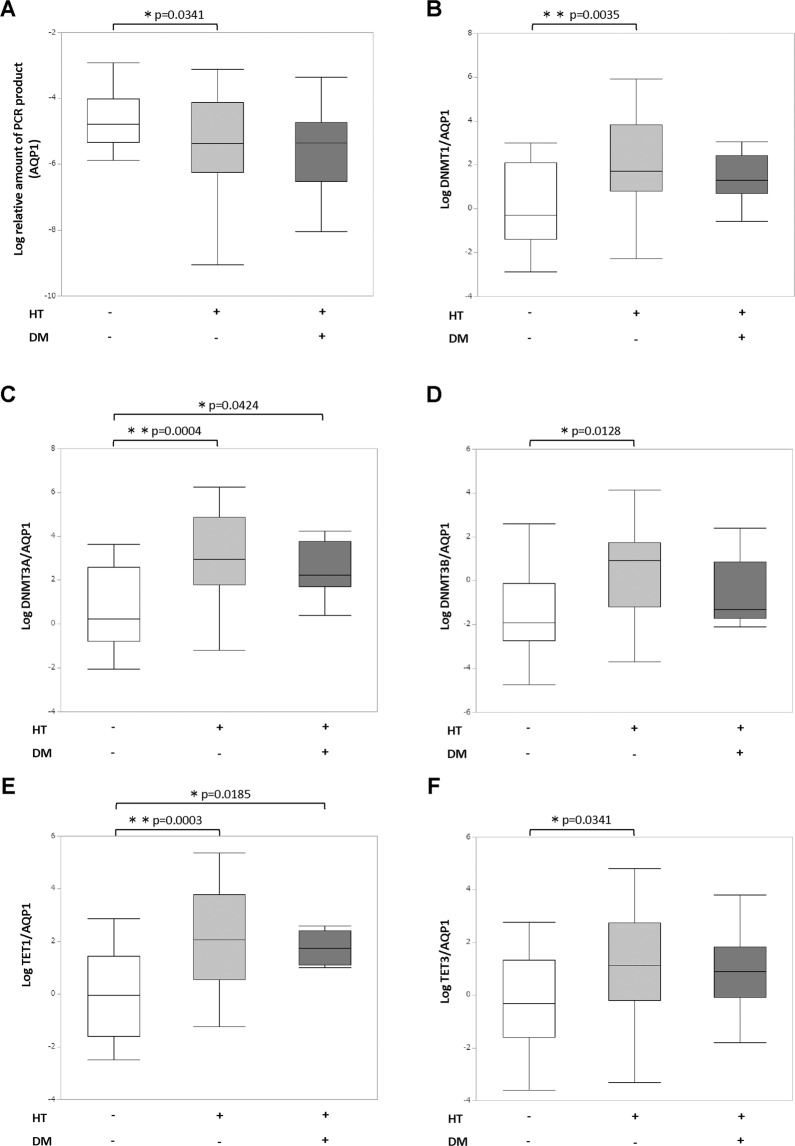
DNA double-strand breaks of the AQP1 gene and expression of DNA methylation modifiers correction for AQP1. (**A**) Detectable increase in DNA DSB sites in the AQP1 gene by the real-time RT-PCR method in patients with hypertension. (**B–F**) Real-time RT-PCR analysis of DNA methylation modifiers correction for AQP1. DM, diabetes mellitus; HT, hypertension.

In patients with hypertension, the long-PCR product of AQP1 gene decreased, which showed increased DNA DSBs of the AQP1 gene in proximal tubular cells (Fig. 2A). We used human cultured podocytes^10^ as negative control, which do not express AQP1. The long-PCR product of the AQP1 gene was significantly increased in the cells since closed chromatin region where transcription is inactive, is less likely to have DNA damage (Supplementary Fig. 1). We also evaluated the urine KAT5/AQP1 which showed no significant differences among patients with or without hypertension. Since it has been reported that DNA DSBs may induce aberrant gene silencing via DNA methylation^11,12^, we next focused on the expression of DNA methylation modifiers. As a result, the expression of DNA methyltransferases DNMT1, 3 A, and 3B and DNA demethylation enzymes TET1 and 3, after correction for AQP1, increased in patients with hypertension (Fig. 2B–F). These results were still significant after age adjustment. We also evaluated sodium-glucose cotransporter 2 (SGLT2) as another specific marker of proximal tubular cells. We excluded two patients who were taking SGLT2 inhibitor in the analysis to exclude the possible effect of the drug to SGLT2 expression. As a result, the long-PCR product of the SGLT2 gene decreased, which may show increased DNA DSBs of the SGLT2 gene in proximal tubular cells (Supplementary Fig. 2A). In addition, the expression of DNA methyltransferases DNMT1, 3 A, and 3B and DNA demethylation enzymes TET1 and 3, after correction for SGLT2, increased in patients with hypertension (Supplementary Fig. 2B–F). Although there was no significant correlation between urine DNMTs/AQP1, TETs/AQP1 and systolic or diastolic BP itself, urine DNMTs/AQP1 and TETs/AQP1 increased depending on the number of antihypertensive drugs, which may reflect the severity of hypertension (Supplementary Fig. 3A–E). There was no significant difference based on the use of specific kinds of antihypertensive drugs.

### Multiple regression analysis of the annual eGFR decline rate

**Figure 3:**
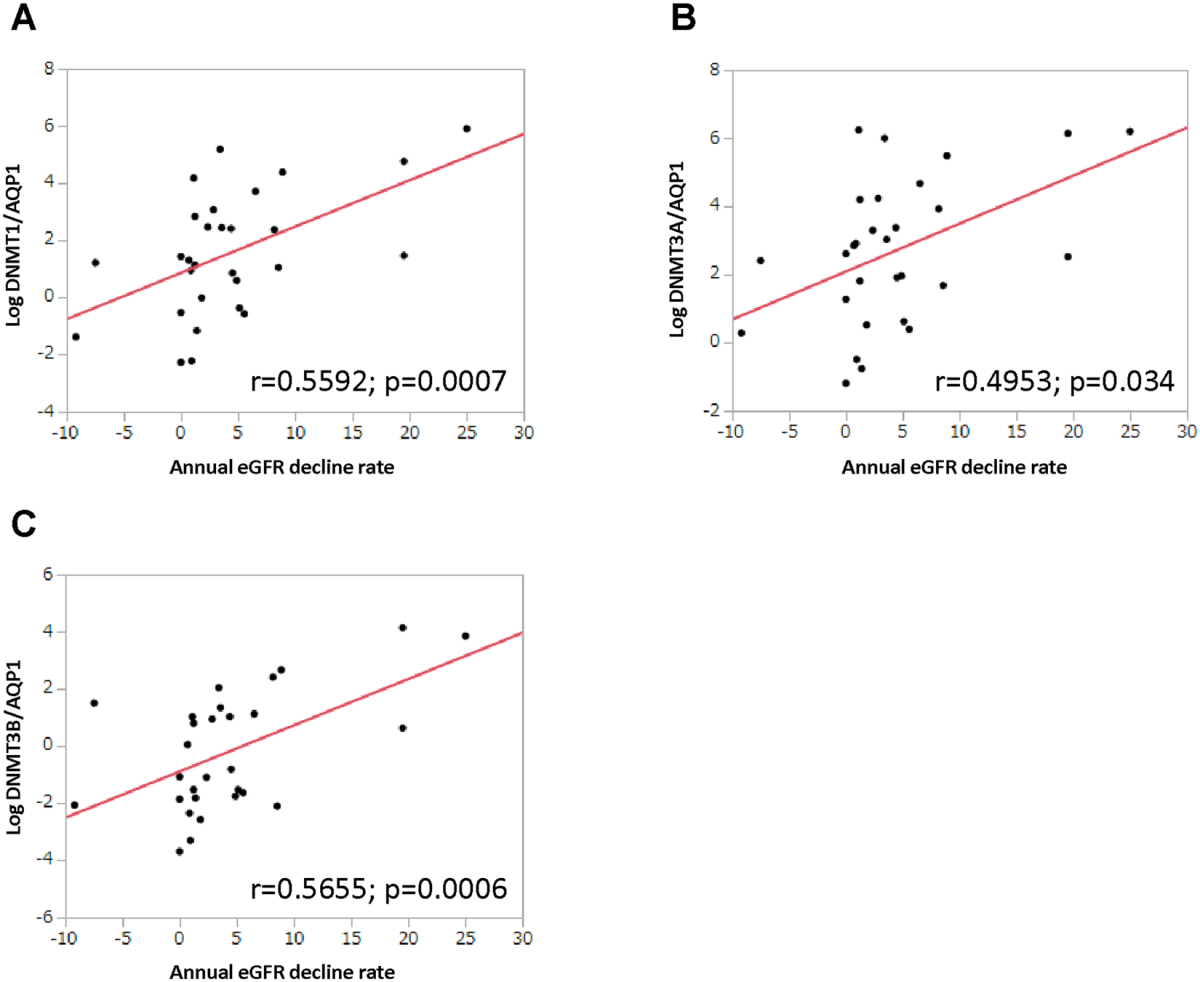
Correlation between urine DNMTs/AQP1 and annual eGFR reduction rate. (**A–C**) Univariate logistic regression analysis showing the association between urine DNMTs/AQP1 expression and the annual eGFR decline rate.

The association between the annual eGFR decline rate and expression of epigenetic modifiers was investigated using univariate logistic regression analysis. It showed that urine DNMT1/AQP1, DNMT3A/AQP1 and DNMT3B/AQP1 were significantly associated with the annual eGFR decline rate (Fig. 3A–C). In addition, they were also significantly associated with the annual eGFR decline rate by multiple regression analysis adjusted for age, baseline eGFR, the presence of diabetes and the amount of albuminuria (Table 2).

**Table 2:** Multiple regression analysis of the annual eGFR reduction rate.

	Log DNMT1/AQP1		Log DNMT3A/AQP1		Log DNMT3B/AQP1	
	Coefficient (95% CI)	p value	Coefficient (95% CI)	p value	Coefficient (95% CI)	p value
Model A	1.46 (0.42 to 2.5)	P = **0.0076**	1.18 (0.16 to 2.19)	P = **0.024**	1.44 (0.45 to 2.42)	P = **0.058**
Model B	1.51 (0.43 to 2.6)	P = **0.008**	1.2 (0.15 to 2.25)	**P** = **0.027**	1.47 (0.45 to 2.49)	P = **0.0065**
Model C	1.19 (0.22 to 2.15)	**P** = **0.0178**	0.98 (0.064 to 1.89)	**P** = **0.0368**	1.10 (0.17 to 2.04)	**P** = **0.0223**

Model A: multiple linear regression model for the annual eGFR reduction rate adjusted by age and baseline eGFR.

Model B: multiple linear regression model for the annual eGFR reduction rate adjusted by age, baseline eGFR and the presence of diabetes.

Model C: multiple linear regression model for the annual eGFR reduction rate adjusted by age, baseline eGFR, the presence of diabetes and the amount of albuminuria.

## Discussion

This study has demonstrated that podocyte DNA DSBs increased with reduced expression of DSB repair factor KAT5 in patients with diabetes, using urine-derived cells. In patients with hypertension, DNA DSBs, primarily in proximal tubular cells, were increased with elevated expression of DNMTs and TETs. Finally, urine DNMTs/AQP1 expression was significantly correlated with the annual eGFR decline rate after adjustment for age, baseline eGFR, the presence of diabetes and the amount of albuminuria.

To evaluate DNA DSBs in the kidney, we performed the quantitative long-PCR method, which was reported previously in mice^2,9^, using genomic DNA extracted from urine-derived cells. It is known that the opened chromatin region, where active transcription is conducted, such as the promoter region of cell-specific marker genes, is vulnerable to DNA damage^13–15^. Therefore, we developed primer sets, including the promoter region of nephrin and AQP1 or SGLT2 that are expressed specifically in podocyte and proximal tubular cells, to assess DNA DSBs in podocytes and proximal tubular cells, respectively. The decreased relative level of PCR products of the nephrin gene, which indicates increased DNA DSBs primarily in podocytes, was observed in patients with diabetes. Moreover, DNA DSBs, primarily in proximal tubular cells, were increased in patients with hypertension. These results suggest that the types of cells prone to DNA damage were different according to disease type, which may cause different renal phenotypes and outcomes. Further examination of various DNA DSB profiles in urine samples depending on each kidney disease may open the possibility of investigating a novel noninvasive marker.

Recently, we have demonstrated elevated podocyte DNA DSB levels with reduced KAT5 expression in murine models of diabetic nephropathy^2^. In the database of Nephroseq (https://www.nephroseq.org), under the name ‘Ju CKD Glom’, are previously reported gene expression arrays performed using human kidney samples from patients with kidney diseases and healthy living donors; compared with normal controls, glomerular KAT5 expression was decreased in diabetic nephropathy, whereas it was not changed in hypertensive nephrosclerosis. This study demonstrated that decreased urine KAT5/nephrin expression, which indicates primarily podocyte KAT5 expression, was observed in diabetes. Of note, the average urinary protein was not increased, and eGFR was not decreased in the population with diabetes compared to that without diabetes, which indicates early-stage nephropathy. In addition, urine KAT5/nephrin significantly reduced in diabetes accompanied with hypertension compared to hypertension alone. Therefore, urine KAT5/nephrin expression may be a possible marker for detecting diabetic alteration in podocytes even in early stages; however, this study has not observed renal outcomes of diabetic patients for long periods.

Emerging evidence has suggested that epigenetic mechanisms, including DNA methylation, are involved in the pathogenesis of CKD and hypertension. The association of global or site-specific DNA methylation with hypertension has been investigated in many studies using blood samples^16,17^; however, the correlation between kidney DNA methylation and hypertension has not been adequately investigated, probably because kidney biopsies are not performed in patients with hypertension alone unless complications of kidney diseases are indicated. The present study indicates that, especially in proximal tubular cells that express AQP1, modulation of DNA methylation through DNMTs may be conducted actively in patients with hypertension, although the DNA methylation status itself has not been examined in this study. A recent *in vivo* study has also shown that increased DNA methylation, which may be induced mainly by DNMT3A, was detected especially in the outer medulla of the kidney in a rat model of salt-induced hypertension^18^. In addition, DNA DSBs, primarily in proximal tubular cells, were also increased in hypertension, which may be one of the causes of elevated expression of DNMTs and TETs because they have roles in the DSB repair process^19,20^. Previous *in vivo* and *in vitro* studies have also shown that angiotensin II, which is one of the major causes of hypertension, induces DNA DSBs in renal cells^21^. These results suggest that hypertension may cause DNA DSBs and modification of DNA methylation primarily in proximal tubules, which indicates a novel strategy for protecting kidneys from hypertensive complications.

Recently, the importance of kidney site-specific DNA methylation on renal function has been demonstrated in humans^22,23^. The present study has suggested that urine DNMTs/AQP1 was correlated with the rate of eGFR decline over one year, indicating that not only altered DNA methylation itself but also altered expression of DNA methylation modulators may be associated with disease progression.

This study has some limitations. First, this study did not follow the renal outcomes for long periods of time. Second, the population with diabetes alone was too small to evaluate the association of DNA DSBs and expression of epigenetic modulators with renal function and outcomes in diabetic nephropathy. Further studies are necessary to determine the significance of DNA DSBs and their related factors in urine-derived cells on renal outcomes in larger populations for extended observational periods.

Despite these limitations, this study suggests the association of kidney DNA DSBs and their epigenetic modifiers with hypertension and diabetes. Detection of kidney DNA DSBs and DNA methylation modulators noninvasively may become a novel strategy for evaluating present renal damage and predicting outcomes.

## Methods

### Study population

Individuals aged 29–93 years old, who visited the outpatient department of nephrology and hypertension at the Keio University Hospital from May 1, 2018 to July 30, 2019, were enrolled. 19 healthy volunteers were included as controls. Patients with hypertension, diabetes, dyslipidemia, hyperuricemia, CKD or asymptomatic hematuria were included in this study. We excluded participants without essential data, including age, sex, body mass index (BMI), systolic blood pressure (BP), diastolic BP and serum chemistry profiles. Individuals who were on renal replacement therapy were also excluded. In total, data from 75 participants (47 males, 28 females) were included and analyzed.

### Clinical evaluation and laboratory measurements

Blood pressure was measured on the right upper arm after subjects had rested at least 5 min in a sitting position in the hospital. Blood pressure was measured with an automatic device (BP-900) with the combination of the Korotkoff sounds method and oscillometric technique (TANITA Co. Tokyo, Japan). Blood and urine samples were collected at the same visit. Blood samples were collected and immediately analyzed using standard hospital laboratory techniques in Keio University Hospital. Urinary protein excretion was calculated from the urinary protein concentration/urinary creatinine concentration at the time of outpatient visit.

### Definitions

eGFR was calculated using the following equation: eGFR (mL/min/1.73 m^2^) = 194 x serum creatinine (−1.094) x age (−0.287) x 0.739 (if female)^24^. Hypertension was defined as systolic BP ≥ 140 mmHg and/or diastolic BP ≥ 90 mmHg or the use of antihypertensive drugs. Diabetes was defined in accordance with the guidelines of the American Diabetes Association as a fasting glucose concentration ≥ 126 mg/dl, HbA1c level ≥ 6.5%^25^ or the use of antihyperglycemic drugs. We calculated the annual eGFR decline rate from the difference between eGFR one year before and after at the time of urine sample collection, which were two years apart.

### Urine sample collection

Fifty milliliters of urine samples were prospectively collected from the outpatients who signed a consent form at the clinic visit. The samples used were the urine left over from routine urine collections, which were midstream samples. All samples were assigned a study number that connected them to clinical information from the chart to deidentify them. RNA extraction and real-time RT-PCR analysis was performed on 45 mL of urine, and 5 mL of urine was used for the DNA DSB analysis.

### Urine processing

Urine was centrifuged at 4 °C for 15 min at 3,500 rpm, and then two 2 mL of the supernatant were removed for measurements of protein and creatinine. The urine pellet was suspended in 500 μL of cold diethylpyrocarbonate- treated PBS (pH, 7.4) at 4 °C. A second 500 μL of PBS was added to wash the bottom of the 50 mL centrifuge tube to recover the remaining pellet material. The transferred pellet material in 1.0 mL PBS was centrifuged at 12,000 rpm for 5 min at 4 °C, and the pellet was used for RNA or DNA analysis. RNA was extracted using ISOGEN (Nippon Gene, Japan), and DNA was extracted with the NucleoSpin Tissue kit (Takara, Japan) according to the manufacturer’s instructions.

### Real-time quantitative PCR

RNA was reverse transcribed using TaqMan Reverse Transcription Reagents (Invitrogen). The real-time RT-PCR reactions were performed using SYBRgreen PCR Master Mix (Applied Biosystems) and run using StepOne devices from Applied Biosystems (Thermo Fisher Scientific). Each measurement was performed as duplicate and the PCR data were normalized to GAPDH gene expression and the comparative Ct quantitation method was used. Primers are listed in Supplementary Table 2.

### Long distance-PCR

The quantitative long-distance PCR method for detecting DNA DSBs is based on the assumption that DNA with fewer DSB lesions will amplify to a greater extent than more damaged DNA if equal amounts of DNA from different samples are amplified under identical conditions, as described previously in mice^8,9^. We created primers to amplify an approximately 10000-bp section of the promoter region of the cell-specific marker genes, as described in Supplementary Table 3. The real-time RT-PCR reactions were performed using SYBRgreen PCR Master Mix (Applied Biosystems) and run using StepOne devices from Applied Biosystems (Thermo Fisher Scientific). The amount of PCR product was evaluated by the standard curve and adjusted by the level of the GAPDH gene. We used human cultured podocytes as a negative control of DNA DSB evaluation of AQP1 gene. The cell line was provided by Dr. Moin A. Saleem (University of Bristol, UK).

### Statistical analysis

For univariate analyses, analysis of variance (ANOVA) followed by Scheffe’s post hoc test and Pearson’s test were used for categorical and continuous variables, respectively. Continuous variables were expressed as the mean ± standard deviation (SD). Multiple linear regression analysis was performed to evaluate the association with annual eGFR decline rate, adjusted for factors, including age, eGFR, presence of diabetes and urinary protein excretion. We confirmed variables with a variance inflation factor (VIF) below 10, which was 1–2 in this study, to minimize multicollinearity. The significance level for all tests in this study was two-sided 5%. All statistical analyses were performed using JMP version 14 (SAS Institute Inc., Cary, NC, USA).

### Study approval

The Ethics Committee of Keio University School of Medicine approved the present study (approval number: 20180013). Written informed consent was received from participants prior to inclusion in the study. Participants were identified using a number, not their name. It was conducted in adherence with the Declaration of Helsinki. All methods were carried out in accordance with the institutional guidelines of the ethics committee at Keio University (Tokyo, Japan).

## Supplementary information

Supplementary Information.
