# Supplementary material for: DNA damage and expression of DNA methylation modulators in urine-derived cells of patients with hypertension and diabetes
Source: Sci Rep. 2020 Feb 25;10:3377. doi: 10.1038/s41598-020-60420-9 (PMC7042287; doi:10.1038/s41598-020-60420-9)
Supplement: Supplementary file 1 — Supplementary Information. [file 41598_2020_60420_MOESM1_ESM.pdf]

DNA damage and expression of DNA methylation modulators in urine-derived cells of patients with hypertension and diabetes

Supplementary information

Akihito Hishikawa M.D., Ph.D. [1], Kaori Hayashi M.D., Ph.D. [1,*], Norifumi Yoshimoto M.D. [1], Ran Nakamichi M.D. [1], Koichiro Homma M.D., Ph.D.,[2] Hiroshi Itoh M.D., Ph.D. [1,]

[1] Division of Nephrology, Endocrinology and Metabolism, Department of Internal Medicine, Keio University School of Medicine, Tokyo, Japan.

[2]Department of Emergency Medicine, School of Medicine, Keio University, Tokyo, Japan.

Correspondence to:

Kaori Hayashi, M.D., Ph.D.

Division of Nephrology, Endocrinology and Metabolism, Department of Internal Medicine, Keio University School of Medicine

35 Shinanomachi, Shinjuku-ku, Tokyo 160-8582, Japan

Tel: 81-3-5363-3796, Fax: 81-3-3359-2745

Email: kaorihayashi@keio.jp

SUPPLEMENTARY TABLES

Supplementary Table 1. Detection percent of the cell markers and epigenetic modifiers.

	Total	Group A HT(-) DM(-)	Group B HT(+) DM(-)	Group C HT(+) DM(+)	p value A vs B	p value B vs C	p value A vs C
N (%)	75 (100)	19 (25)	41(55)	15 (20)			
Nephrin	37 (49)	17 (89)	15 (37)	5 (33)	**0.0001**	0.8220	**0.0007**
AQP1	50 (67)	17 (89)	22 (54)	11 (73)	**0.0068**	0.1851	0.2203
AQP2	49 (65)	17 (89)	22 (54)	10 (67)	**0.0068**	0.3837	0.1025
KRT20	55 (73)	17 (89)	27 (66)	11 (73)	0.0543	0.5956	0.2203
KAT5	43 (57)	19 (100)	14 (34)	10 (67)	**<0.0001**	**0.0294**	**0.0064**
DNMT1	75 (100)	19 (100)	41 (100)	15 (100)	n/a	n/a	n/a
DNMT3A	75 (100)	19 (100)	41 (100)	15 (100)	n/a	n/a	n/a
DNMT3B	75 (100)	19 (100)	41 (100)	15 (100)	n/a	n/a	n/a
TET1	75 (100)	19 (100)	41 (100)	15 (100)	n/a	n/a	n/a
TET2	41 (55)	17 (89)	17 (41)	7 (47)	**0.0005**	0.7275	**0.0065**
TET3	75 (100)	19 (100)	41 (100)	15 (100)	n/a	n/a	n/a
SGLT2	75 (100)	19 (100)	41 (100)	15 (100)	n/a	n/a	n/a

Supplementary Table 2. Sequence of Real-time PCR primers

**Name**	**F/R**	**Sequence**
GAPDH	F	TGATGACATCAAGAAGGTGG
	R	TTTCTTACTCCTTGGAGGCC
nephrin	F	TGGCGATTCCTGCCTCCGTT
	R	TTCTGCTGGGAGCCCTCGTT
AQP1	F	CTGGGCATCGAGATCATCGG
	R	ATCCCACAGCCAGTGTAGTCA
AQP2	F	GCTCCGCTCCATAGCCTTC
	R	GGGTGCCAATACCCAAGCC
KRT20	F	GGACGACACCCAGCGTTTAT
	R	CGCTCCCATAGTTCACCGTG
KAT5	F	GGGGAGATAATCGAGGGCTG
	R	TCCAGACGTTTGTTGAAGTCAAT
DNMT1	F	CAAACCCCTTTCCAAACCTC
	R	TAATCCTGGGGCTAGGTGAA
DNMT3A	F	CCTGAAGCCTCAAGAGCAGT
	R	TGGTCTCCTTCTGTTCTTTGC
DNMT3B	F	GGAAATTAGAATCAAGGAAATACGA
	R	AATTTGTCTTGAGGCGCTTG
TET1	F	TCTGTTGTTGTGCCTCTGGA
	R	GCCTTTAAAACTTTGGGCTTC
TET2	F	ACGCTTGGAAGCAGGAGAT
	R	AAGGCTGCCCTCTAGTTGAA
TET3	F	CGCCTCTATCCGGGAACT
	R	TCCCCGTGTAGATGACCTTC
SGLT2	F	ACACGGTACAGACCTTCGTCA
	R	GCTGCTCCCAGGTATTTGTC

Supplementary Table 3. Gene-specific primer pairs used for quantitative long-distance PCR

**Name**	**F/R**	**Sequence**
GAPDH	F	GCCCTCAACGACCACTTTGT
	R	GCCAGACCCTGCACTTTTTAAG
nephrin	F	CTGCCATCAGCAACTCTCCA
	R	CTCTGCCTCTGTTGTGCTGA
AQP1	F	TCAATGCAGGGCTGGGTTAG
	R	ACCCCGTTGGACAGGTTTTT
SGLT2	F	GGACTGTGCTAGCTGAAGGG
	R	AACGCAGTTGGTCTTAGGCA

Suppl Fig. 1

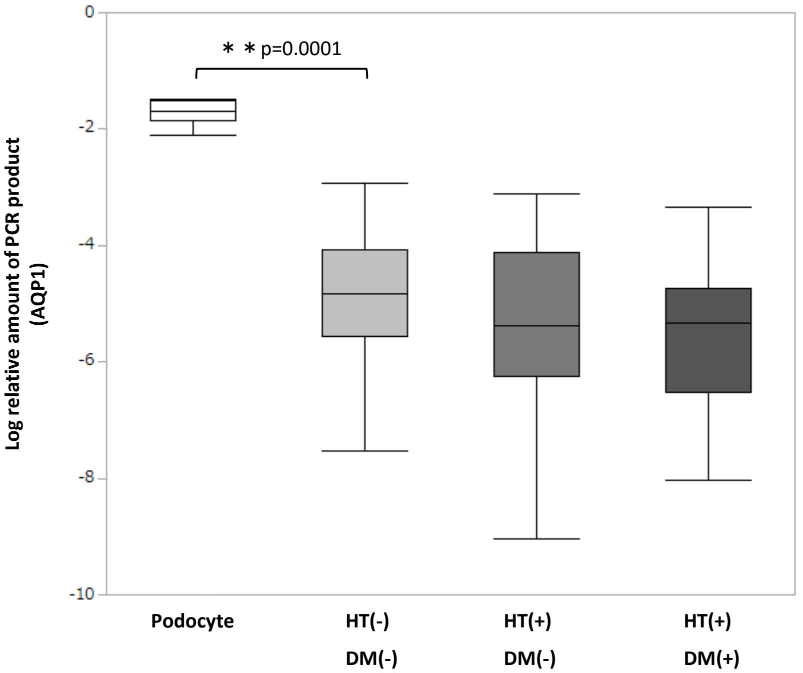

0
-2
-4
-6
-8
-10
Log relative amount of PCR product (AQP1)
＊＊p=0.0001
Podocyte
HT(-) DM(-)
HT(+) DM(-)
HT(+) DM(+)

Suppl Fig. 2

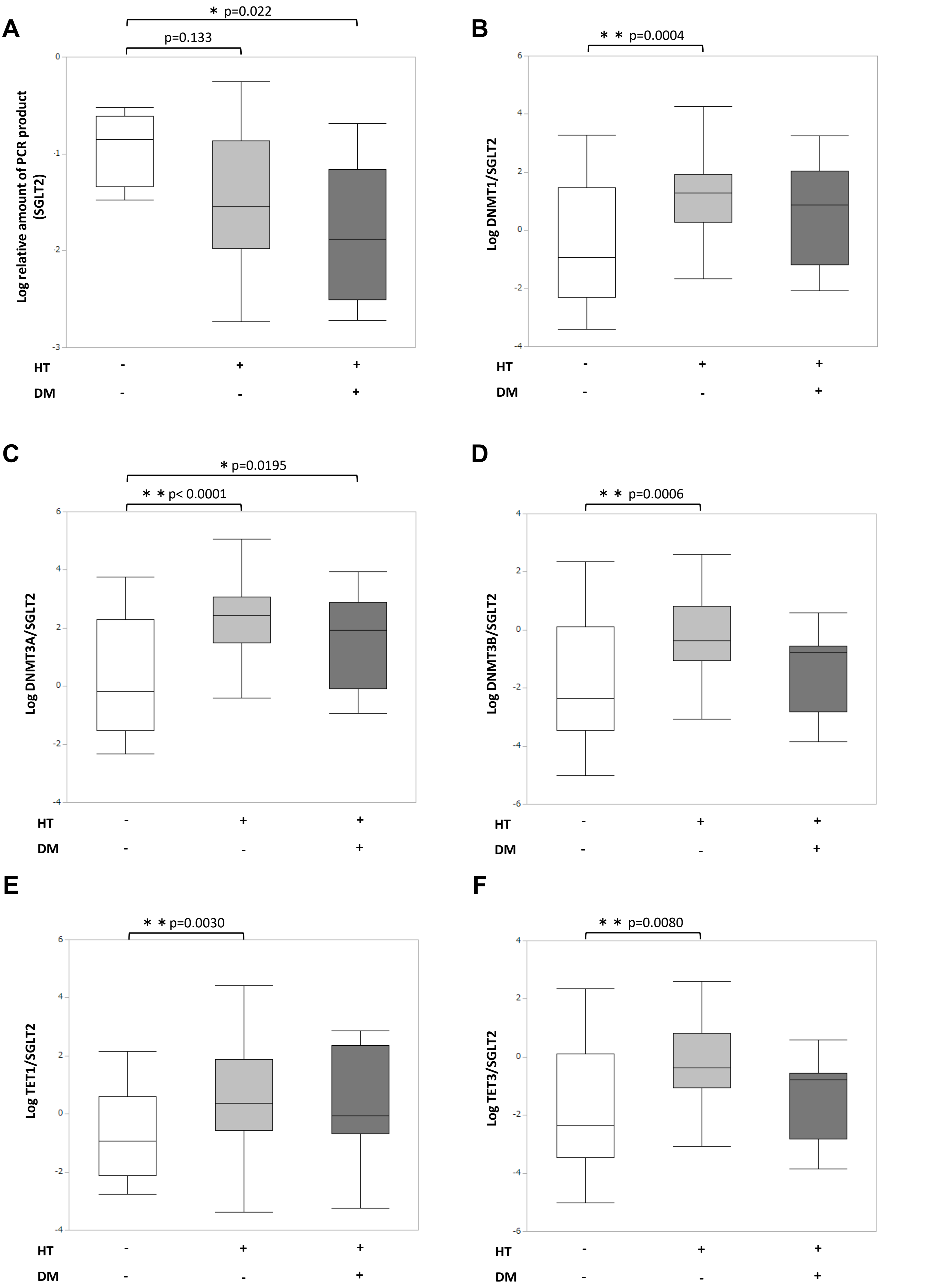
A
* p=0.022
p=0.133
Log relative amount of PCR product (SGLT2)
0
-1
-2
-3
HT - + +
DM - - +
B
* * p=0.0004
Log DNMT1/SGLT2
6
4
2
0
-2
-4
HT - + +
DM - - +
C
* p=0.0195
* * p< 0.0001
Log DNMT3A/SGLT2
6
4
2
0
-2
-4
HT - + +
DM - - +
D
* * p=0.0006
Log DNMT3B/SGLT2
4
2
0
-2
-4
-6
HT - + +
DM - - +
E
* * p=0.0030
Log TET1/SGLT2
6
4
2
0
-2
-4
HT - + +
DM - - +
F
* * p=0.0080
Log TET3/SGLT2
4
2
0
-2
-4
-6
HT - + +
DM - - +

Suppl Fig. 3

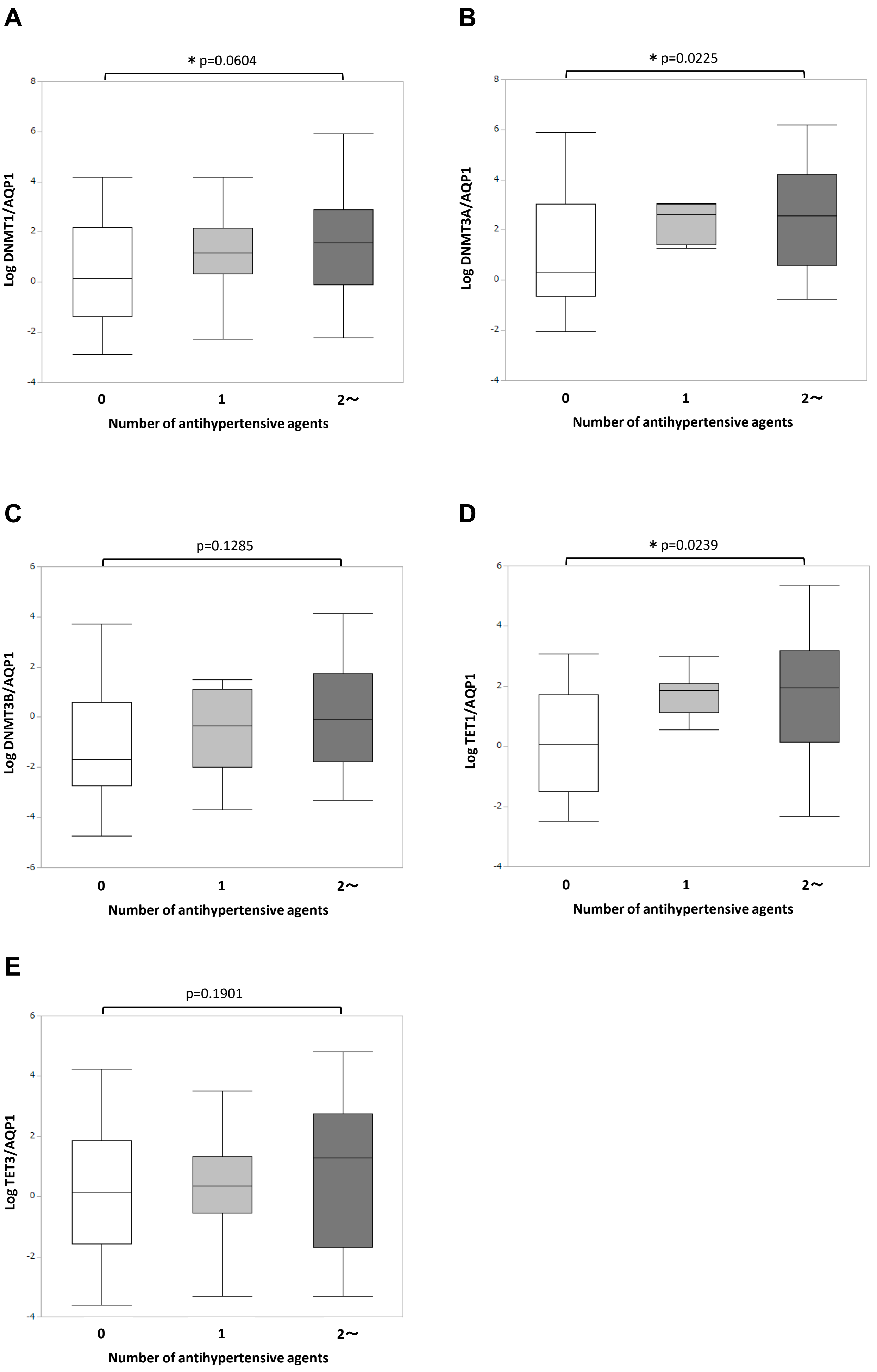
A
＊p=0.0604
Log DNMT1/AQP1
0
1
2〜
Number of antihypertensive agents
B
＊p=0.0225
Log DNMT3A/AQP1
0
1
2〜
Number of antihypertensive agents
C
p=0.1285
Log DNMT3B/AQP1
0
1
2〜
Number of antihypertensive agents
D
＊p=0.0239
Log TET1/AQP1
0
1
2〜
Number of antihypertensive agents
E
p=0.1901
Log TET3/AQP1
0
1
2〜
Number of antihypertensive agents
